# Lived Experiences of Newly Admitted to Long-Term Care Facilities among Older Adults with Disabilities in Taiwan

**DOI:** 10.3390/ijerph19031816

**Published:** 2022-02-05

**Authors:** Nai-Hui Chien, Chin-Hsing Tsai, Hung-Ru Lin

**Affiliations:** 1 Department of Nursing, College of Nursing, Chang Gung University of Science and Technology, Taoyuan 33303, Taiwan; sychien@mail.cgust.edu.tw (N.-H.C.); wimsimtsai@mail.cgust.edu.tw (C.-H.T.); 2 School of Nursing, College of Nursing, National Taipei University of Nursing and Health Sciences, Taipei 112303, Taiwan

**Keywords:** older adults with disabilities, newly admitted to long-term care facilities, lived experiences, descriptive phenomenology, qualitative

## Abstract

This study aimed to explore the lived experiences of Taiwanese older adults with disabilities newly admitted to long-term care facilities (LTCFs). A descriptive phenomenological method was used. Colaizzi’s method analysis of 15 participant interviews revealed six themes: “living here is a last resort”, “I don’t like it but still have to live here”, “my needs are not understood”, “looking forward to emotional support”, “practicing the way of survival”, and “trying to make myself better”. The older adults were admitted to the LTCF as they or their family members could not take care of themselves due to their disability. Participants explained their new life in the LTCF was like a prison, it was not easy for their needs to be understood. They used self-adjustment and established relationships with staff in the LTCF in order to live a stable life. They lived their lives with silence and alertness to practice the way of survival. They strived to make themselves better through rehabilitation, taking good care of their bodies, and finding their focus and value of life. It is important to pay attention to the care needs as well as life adjustment problems for newly older adults with disabilities in order to assist them in opening up new life experiences in LTCFs.

## 1. Introduction

Population aging is a global trend. In 2020, the proportion of older people over 65 in Taiwan reached 16.07%, and it is expected to become a super-aged society when its older population reaches 20% by 2026. The population aging rate in Taiwan has surpassed that of European and American countries, and the weakness, disabilities, and long-term care issues that accompany the rapidly growing older population are gaining increasingly more attention [1]. According to a survey of the status of older adults in Taiwan [2], 28.16% of older people over 65 years old have at least one difficulty in the activities of daily living (ADLs), and 26.66% of them have at least one difficulty in the instrumental activities of daily living (IADLs). In terms of living arrangements, the older adults in LTCFs account for about 1.22% of the older population, and 56% of older adults think that the most ideal way of living is to live with their children or family members. When the older people are unable to take care of themselves due to disability, 35.2% are willing to live in LTCFs. In addition, 74.2% of older adults’ admission to LTCFs is due to being disabled and needing care. Long-term institutional care is becoming an alternative to the family care model, and the rate of older adults with disabilities being forced to move to LTCFs is gradually increasing [3,4,5].

New admission to LTCFs is an overwhelming life change for the older adults with disabilities [6], having significant impact on the health and quality of life of older adults. Research has shown that there are many aspects of adaptation problems in the life of older adults with disabilities in LTCFs, including low self-esteem, feelings of loss, feelings of unfamiliar environment, and alienation; fixed time for work and rest; anxiety, loss of self, loneliness, grief, insecurity, and withdrawal; or other migration stress syndromes [7]. Especially during their first year after moving in, the older adults experience more serious psychological anxiety than residents in LTCFs for a longer term. The new pressure of living in a LTCF can significantly predict the occurrence of depression, which is a risk factor for residents [8]. Melrose [9] found that most of the psychological effects on the older adults moving to LTCFs emerged within the first 6 months, and such effects varied depending on the physical and psychological state of the older adults.

Older adults with disabilities must face the process of adapting to life in a LTCF whether they are admitted voluntarily or involuntarily [2,10]. Disability itself is a source of stress for older adults, and when they are in a state of physical and mental exhaustion, their admission to a LTCF brings new challenges. Their disability coupled with the pressure of being newly admitted may cause older adults to become more debilitated and disabled, which may further damage their original coping ability. Moreover, the poor attitudes and ethics of the professionals in the LTCFs, standard regulations, physical weakness, and high dependence will all hinder the adjustment process of new residents [11]. Fitzpatrick and Tzouvara [12] stated that moving into a LTCF is an important life event for the older adults; thus, LTCF staff must understand the factors that promote and hinder relocation adaptation from different perspectives and provide a series of individualized care services and various resources to support the older adults to healthily adapt to long-term institutional life after moving in. If staff understand the past lifestyle and cultural needs of the older adults, this will help in alleviating their anxiety, promoting their adaptation to their new life in the LTCF, and regarding the LTCF as their “last home” [13].

Johnson and Bibbo [14] used a phenomenological research method to interview eight older adults regarding their lived experiences after they moved into a LTCF. They found that the older adult residents did not consider the LTCF as “home”, and they believed that life in the LTCF was beyond their control, and with limited autonomy; however, they took positive attitudes towards adjusting and better adapting to the new environment. To understand the experiences of the older adults relocated to Taiwan’s LTCFs, Tsai et al. [10] utilized the grounded theory research method to interview 15 older adult participants, finding four interrelated themes: the hope to reduce the burden while keeping in touch with family members; the recognized obstacles to adaptation; valuing tailor-made care; and acceptance and participation. These four themes influenced each other and reflected the mood of the older adults at different stages of their journey after moving into the LTCF.

Some scholars analyzed the impacts of cultural factors on older Americans and Egyptians living in LTCFs in the United States, finding that American older adults were better than Egyptian older adults in their readiness to move, having independence in daily life, feelings of health, adaptability, and their ability to move; thus, cultural factors affected the older adults’ mode and experience in dealing with moving [13,15]. In Japan, which has a culture relatively similar to that of Taiwan, older adults will reflect on their living conditions after entering a LTCF; clarify and evaluate the positive and negative experiences in their life in the LTCF; and slowly accept through the process of repeated thinking, finding meaning, and developing skills in response to their life in the LTCF [16].

Studies have shown that when the ideal and actual living arrangements of the older adults are the same, it has a positive and significant impact on their overall life and satisfaction [17]. Being newly admitted to a LTCF forces older adults with disabilities to leave their homes and face a completely new life pattern, which is a major life change that requires attention [5,8,10,18,19]. In Taiwan’s traditional concept, home is the most anticipated residence for the older adults. In Taiwanese society and culture, older adults want to live with their children, as they hope to have family happiness living among their children and grandchildren, yearning for a life of “peaceful old age”.

Moving into a LTCF may greatly change the old-age care culture, making it difficult for them to adapt to their new life in a LTCF. To date, limited knowledge is present regarding how older adults in Taiwan develop under the traditional culture view and in explaining the experience of individuals newly admitted to the LTCFs due to disability. The results of this study can help the staff of LTCFs to provide appropriate care for newly admitted older adults with disabilities. Therefore, the purpose of this study was to explore the lived experiences of newly admitted to LTCFs among older adults with disabilities in Taiwan.

## 2. Materials and Methods

### 2.1. Design

A qualitative research design was used to explore the lived experiences of newly admitted to LTCFs among older adults with disabilities in Taiwan. The descriptive phenomenological research method using in-depth interviews was chosen to gather information on the subjective essence of each individual’s experience. The descriptive phenomenology method involves “direct exploration, analysis, and description of particular phenomena, as free as possible from unexamined presuppositions, aiming at maximum intuitive presentation” (p. 57, [20]). It stimulates our perception of lived experience while emphasizing the richness, breadth, and depth of those experiences (p. 70, [20]).

### 2.2. Sample and Setting

A purposive sample was used to gather data in this study. The inclusion criteria for participants were the following: (1) aged 65 years or older; (2) admitted to the LTCF due to their disability, with ADL scores below 75 points; (3) within 1 year of admission to the LTCF; (4) ability to communicate or express clearly in Mandarin or Taiwanese; (5) no obvious cognitive impairments, with normal cognitive function (MMSE 18 points or more); (6) consent to participate in this study. Potential participants would be excluded if their medical condition precluded participation in an interview session lasting 30 min. Participants were drawn from 2 different LTCFs in northern Taiwan, both of which accommodated residents with varying degrees of disability. One accommodated 120 and the other accommodated 350 older adults with disabilities. The location for interviews, chosen by the participants, was either in a private room or in a quiet corner at the LTCF, whichever was the most comfortable, relaxing, and without interference for them.

The sample size for the qualitative study was based on the concept of saturation, which refers to the repetition of discovered information and confirmation of collected data [21]. It is recommended that long interviews with up to 10 people be conducted when choosing the phenomenological research method [22].

### 2.3. Data Collection and Analysis

Permission for this study was obtained from the institutional review board of a medical foundation located in northern Taiwan (IRB number: 202001224B0). Before the formal closure of participant recruitment, researchers had four weeks of daytime field observations in the LTCFs to familiarize themselves with the LTCF environment and the possible participants. Afterwards, the social workers in the LTCFs first introduced the study to the older residents, and then the researchers further explained to the individuals with preliminary intention to participate in the study. The researchers attended the LTCFs and contacted the potential participants for at least 2 days before the participants were recruited. After the trust relationship was established, the researchers explained the research purpose and started the interviews after obtaining the signed consent form. The semi-structured interview guidelines (Table 1) that were used to conduct one-on-one audio interviews were created after discussions with 3 senior researchers who specialize in qualitative study, older adult care, and long-term care. Before the data were collected, a pilot study was performed to confirm the appropriateness of the interview guideline. Interviews lasted approximately 60 min each, were audiotaped with the participant’s permission, and were transcribed verbatim immediately after the interview.

**Figure 1:**
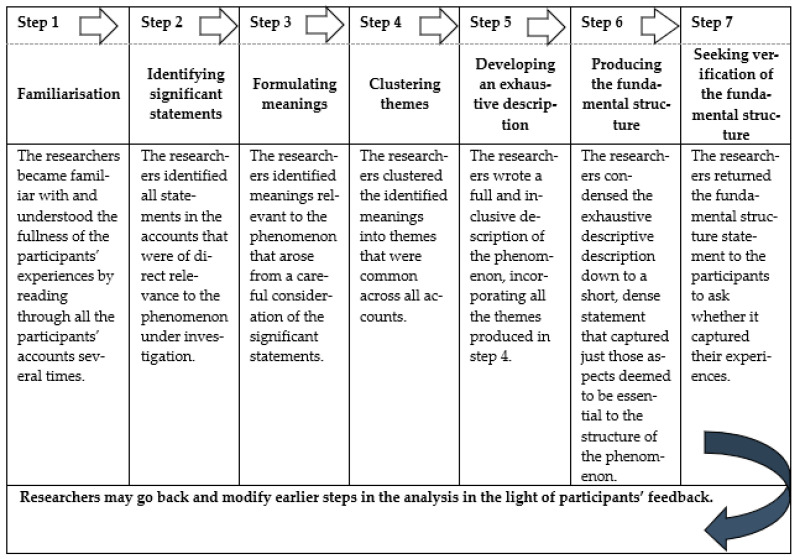
Steps of Colaizzi’s descriptive phenomenological data analysis method.

Data collection was ended when saturation was achieved; that is, no new themes emerged from the participants’ narratives and the data were becoming repetitious [23]. In addition, during each interview, the interviewer recorded the participant’s non-verbal behaviors, any special events that occurred, and thoughts derived from the interview. This study conducted data analysis with Colaizzi’s analysis method [21], which included 7 steps (Figure 1). The objective of step 7 was to validate the preliminary analysis with the participants, in which each participant was asked to validate if the statements, ideas, and words reported by the researchers illustrate their experience of newly admitted to LTCF. This approach was chosen because it allows descriptions of phenomena as experienced in life and aims to offer an understanding of the internal meaning of a person’s experience in the world. Thus, a comprehensive, culturally competent understanding of the phenomenon was obtained.

### 2.4. Trustworthiness

Trustworthiness of the data was established by using the criteria of Lincoln and Guba: credibility, transferability, dependability, and confirmability [24]. All researchers in this study had been trained for qualitative study and were experienced in aged care for more than 10 years. Before the interview, the researcher spent about two days establishing trust relationships with the older adults in the LTCF. The data were collected by open-ended and in-depth interviews in which participants could describe their subjective experiences in detail. The recording of the interview was transcribed verbatim within 72 h after each interview was finished. The typed transcripts were carefully checked by listening to each type again to ensure the accuracy of the transcripts. In addition, participants were asked to validate the findings to establish credibility. The method of peer debriefing was adopted to improve the dependability of data analysis. In terms of transferability, the participants recruited in this study were from various backgrounds; this quality increased the breadth of data and possible application of these findings to other older adults with disabilities newly admitted to LTCFs in future. In terms of dependability, the first author conducted all interviews to maintain consistency. A peer review analysis process was also used to validate the dependability. Three senior researchers who specialized in long-term care, care of older people, and qualitative studies completed the data analysis of the verbatim text alone and then cross-examined the analysis. For confirmability, the researchers faithfully and completely translated the interview content recordings into text and added memos and field notes regarding nonverbal behaviors to enable the “audit trail” in the thinking and context of the data analysis. The researchers examined their own perceptions towards the research questions and continued to reflect on them throughout the research process to minimize any possible personal bias. In addition, the data obtained were marked with times and dates and properly stored for future reference.

### 2.5. Ethical Consideration

This study was conducted after being reviewed and approved by an institutional review board in northern Taiwan. Besides written information provided during recruitment, the purpose and process of the study, including the participant’s right and protection of identity, were discussed, and a signed consent form was obtained prior to the interview. During the interview, when participants raised sensitive issues and information, support was provided, and maintenance of confidentiality was reassured. In addition, in order to protect the identity of the participants, the researchers used letters (A–O) instead of the participants’ real names in presenting the documents or reports of this study.

## 3. Results

Fifteen older adults with disabilities newly admitted to the LTCF participated in comprehensive interviews, specifically, eight males and seven females, aged between 65 and 90 years old, with an average age of 73.5 years old, with the scores of the ADL Scale for Disability Level ranging from 35 to 70. The reasons for admission into the LTCF were mostly the difficulties of care at home and the lack of caring capacity after the disability. The older adults’ time of stay in the LTCF was between 3 and 9 months, as shown in Table 2. Data analysis of the interviews revealed six themes: “living here is a last resort”, “don’t like it but have to stay”, “needs not understood”, “hope for emotional support”, “practicing the way of survival”, and “striving to be better”. Each themes contained two to three sub-themes, as shown in Table 3.

### 3.1. Living Here Is a Last Resort

The older adults with disabilities said that the decision to move into the LTCF was a last resort. They chose to be admitted into the LTCF because they lost the ability to take care of themselves and could not be taken care of at home. Due to inconvenience, they could not go home and had no choice but to settle here.

#### 3.1.1. Difficulty Being Cared for at Home

In this study, all of the 15 older adults mentioned that they were unable to take care of themselves due to disability and needed others to assist in daily life; however, the family cannot provide adequate care manpower and resources, and thus the older adults with disabilities had to move to the LTCFs to live. For example:

“I accidentally fell and injured my feet. After surgery, I couldn’t stand up and needed someone to hold me. I needed help in my life. I had difficulty moving around at home, and no one could take care of me all the time. I had no choice but to come here.”

(Participant C)

#### 3.1.2. Hard to Get Home

This study found that the family members of some older adults with disabilities arranged for them to admit into LTCFs against their personal wishes. Nine of the 15 participants have mentioned that they were always thinking about home after they admitted, would not let go of everything from their previous life at home, and wanted to go home, but they had no choice but to remain in the LTCF, as they have difficulty moving after becoming disabled. They cannot go home without help and lamented the hard way home. For example:

“My home is on the 5th floor, and it’s an apartment without an elevator. If I live at home, I have to bother my eldest daughter to find someone to lift me up to my home on the 5th floor. Because I can’t walk on my feet now, I can’t climb up by myself.”

(Participant I)

### 3.2. Don’t Like It Here but Have to Stay

After the older adults with disabilities admitted to the LTCF, they felt the dilemma of life being disturbed, no privacy, no freedom, and their needs not being immediately met; however, they knew that the LTCF was necessary, and they helplessly told themselves that, while they do not like it, they still need to settle down.

#### 3.2.1. Life Here Is Like in a Prison

In this study, the participants obviously felt that their new life in the LTCF made them feel uncomfortable, as their life must be carried out according to the LTCF’s life schedule, and they must cooperate with the habits of others or cooperate with participating in the activities of the LTCF. In addition, mobility was restricted and there was no freedom to enter and exit the LTCF at will. Particularly, seven of the participants mentioned that life in an LTCF is like living in a prison, and it is uncomfortable; for example:

“I usually can’t go in and out freely here. It’s very troublesome to go shopping. When I go out, I need family members or friends to pick me up. I’m alone, how can I have my relatives come. This restricted life here is like a prison (with tears in his eyes, and hand wiping tears). I used to sneak out and didn’t come back, so they called the police to look for me.... I really feel sad, and I can’t get out by myself if I want to go out and get some air.”

(Participant M)

#### 3.2.2. The Body Here but the Heart Is Not

In this study, 8 of the 15 older adults with disabilities reported that after they were admitted to the LTCF, although they lived in the LTCF, their hearts were not there at all, and they could not know everything about their family members while in the LTCF. They were concerned about their family members, property, or house, and worried about the house while they are away. Even if the children were already independent adults, they still cared about their homes. For example:

“I have worked hard for a lifetime and have some savings. I am afraid that my children cannot handle these savings. My seal information is kept by my children, and I am afraid that others will cheat on my property.”

(Participant B)

#### 3.2.3. Living Here Helplessly

With the passage of the time after admission, the older adults slowly realized that they still could not return home due to their continuous disability. Although they did not like it, they needed to be taken care of because of their disability and were unable to live independently; thus, they could do nothing but continue to live here. In this study, eight of the participants mentioned this experience, for example:

“No old man likes to live here. It is more comfortable to live at home! If you are to live here, would you like it? Living here is really not good (eyes opening wide), but I just can’t move easily, so I can do nothing!”

(Participant D)

### 3.3. Needs Not Understood

The participants hoped that the LTCF would shorten the waiting time for their requirements to be met during their care, and the LTCF would understand their situation, meaning they cannot take care of their own needs, and hoped staffs would consider the older adults’ needs as their own needs.

#### 3.3.1. Demand Cannot Be Met Immediately

In this study, eight of the older adults reported that when they need to be taken care of, although they know that the staff is busy with other things and could not deal with their problem immediately, they wanted to their needs to be satisfied as quickly as possible due to discomfort. For example:

“When I require something, the staffs taking care of me would not reject me, but they are busy with their own affairs or other people’s affairs, so you always have to wait after you call for help. Sometimes it takes a long time to wait someone comes to help me. At the time, I can’t sit and I can’t sleep, because no one can take care of me as soon as possible. It’s particularly uncomfortable when my diaper is wet!”

(Participant N)

#### 3.3.2. Hope to Be Cared for with Sympathy

Older adults with abilities who have admitted to the LTCF have many things in daily life that cannot be taken care of by themselves. Eleven of the participants in this study expressed that they hope that staff will take a more compassionate attitude to provide care to understand the inconvenience and powerlessness of people with disabilities. For example:

“Some staff are very careful, but some are very rude! The staff must understand me in terms of care. I understand that for a big man like me, it is inconvenient to take care of in many aspects (referring to changing diapers or bathing), but as a person with disability needs to be taken care of by others. They should try to stand in others’ shoes, so the person being taken care of will be in a better mood. I have to say, I do not like to be taken care by others for things like bowel movements and urinating!”

(Participant F)

### 3.4. Hope for Emotional Support

The older adults with disabilities who live in an LTCF know that they cannot return home temporarily. In the process of adapting to their relocation, they slowly develop interactions with others in the LTCF and establish new relationships and find emotional attachments and support from their new relationships, including the staff in the LTCF, other residents, and even intern students. They received emotional support and satisfaction from their company or care process.

#### 3.4.1. Love Family to Visit

After the older adults with disabilities were admitted into the LTCF, they counted the days every day and looked forward to family visits. In this study, 11 participants mentioned that family visits have become the biggest pillar of their life in the LTCF, for example:

“I like that the children bring my favorite food when they come here (talking with a smile and raised eyebrows, and the corners of the mouth). They would push my wheelchair for a walk outside or sunbathe for a while. They chat with me and let me know the situation at home.”

(Participant D)

#### 3.4.2. Like to Be Accompanied

The participants in this study were residents of long-term care teaching facilities. In the interviews, it was found that intern students play an important role in accompanying the older adults in the LTCF, and the companionship of the students made the older adults feel the emotional support as from their grandchildren. Nine of the participants expressed this experience, for example:

“I think the students are so cute. They accompany me like my grandson. I like it that they care for me so much and have more time to accompany us to talk. Sometimes I have no one to talk with all day. If there are students to chat with, we can say anything to them. They often stay with us and care about us from time to time.”

(Participant C)

#### 3.4.3. Looking Forward to Building Relationships with Residents

Establishing relationships with others is an important indicator for the older adults to adapt to life in LTCF. After they realize that they must continue to live in the LTCF, they expect to start a new life and connect with others around them. Twelve of the participants mentioned this experience, for example:

“When I first came here, I was not familiar with other people, and I am not very free to move. It is not easy to get along with other people, but I slowly found residents from the same province. I hope that I can make friends with them and take care of each other.”

(Participant A)

### 3.5. Practicing the Way of Survival

Older adults with disabilities have a deep understanding that living in an LTCF is not same as living at home. When faced with setbacks and difficulties, they adopted a silent attitude and spoke less to seek good fortune and avoid evil, leaving their grievances behind. They always reminded themselves to be alert, pay attention to interactions with others, and learn to find joy amid hardship in order to be a good resident that people like in exchange for their living space in the LTCF.

#### 3.5.1. Staying Alert and Remaining Silent

The older adults living in the LTCF have their own different backgrounds. After their admission to the LTCF, while they got along with other residents day and night, they sometimes had suspicions or frictions with each other. In order to adapt to the people, things, and environment in the LTCF, one of the adaptation skills commonly used by new residents in LTCFs is that they adopt a cautious and vigilant attitude to observe and respond to things related to them. In addition, in order to live a safe and peaceful life in the LTCF, they chose to respond to conflict situations in a silent manner. Seven of the participants reported this experience, for example:

“I avoid arguing with others. If I really can’t bear it, I will get out of the way, go to other places to see the scenery. If I must interact with others, I choose to “seek good fortune and avoid evil” and try to ignore it. But, no one can understand what life is like with this kind of loneliness?”

(Participant B)

#### 3.5.2. Trying to Have Fun in Life

Seven of the participants said that although they felt lonely and helpless after entering the LTCF, that they did have people to take care of their everyday needs in life. Thus, they began to change their minds, found the kinetic energy to support their continued life in the LTCF, had fun in life, and adapted to life in the LTCF; for example, a participant jokingly said:

“In Chinese tradition, only the emperor can be served by others. I don’t need to prepare anything here, yet I can live here, and eat and sleep, and someone will even help me deal with incontinence. I just consider being taken care of as being served! We have to believe it to be a pleasure to continue to live here.”

(Participant E)

#### 3.5.3. Being a Good Resident

As living in the LTCF became longer and longer, the older adults found a strategy that allows them to live better, which is to make themselves a good person to take care of. This phenomenon is a state of mind that oneself will be good if others feel good. Ten of the participants expressed this viewpoint, for example:

“To live here, you must make yourself a person that can be easily taken care of. You must think about ways to solve problems, to calm your emotions. You must pay attention to your diet as much as possible. Don’t eat anything you like, so that you don’t need to go to the bathroom all the time, or use the toilet less frequently, as you can’t ask the staff to help you all the time, right?”

(Participant H)

### 3.6. Striving to Be Better

After experiencing the psychological struggles of living in an LTCF, the older adults with disabilities began to try to keep their body and mood in a better state. Some residents even hoped that their physical condition will continue to improve, and one day they can leave the LTCF and return to their home.

#### 3.6.1. Finding a Way to Keep the Health from Going Bad

In this study, eight of the older adults described they realized that their disability was getting worse with aging and the course of the disease, and they tried to keep their bodies from getting worse, telling themselves to move as much as possible on their own. For example:

“Find a way to prevent my body from getting worse because of getting older and older. When getting older and the body cannot stand it, it will eventually break down. So, I should move by myself as much as I can, when I am here.”

(Participant J)

#### 3.6.2. Participating in Active Rehabilitation for Progress

Nine participants in this study had the same expectation, that they would work hard to improve their disability, and even hoped that one day they could go back home. For example:

“My everyday life here is to match the work and rest. The focus of a day’s life is rehabilitation. Rehabilitation is very important to me. Rehabilitation makes me better and better. I hope I can walk. I hope that one day I can rebuild my body and get better. If I don’t have to live here anymore, I can walk home, and I will become the healthy and comfortable me, as before.”

(Participant G)

#### 3.6.3. Trying to Find the Focus and Value of Life

Facing life in LTCF, the participants slowly began to change their mindsets. They described that they must arrange their own life there and start to learn how to live and find the abilities they once possessed. They wanted to live according to their own values and functions, find a focus of life, and develop a completely different life experience in LTCF. Eight participants expressed this viewpoint. For example:

“I used to be a caregiver. I live here, but I have better physical functions than others. I help take care of other residents to convey emotions and find my own abilities and values. I can help others here, and I especially regard taking care of the older adults next door as my responsibility, so I have more focus in life.”

(Participant O)

## 4. Discussion

In this study, older adults believed that being admitted to the LTCF due to disability caused great hardship. However, despite their difficulties, older adults could find ways to better adapt to life in LTCF even after confronting very difficult situations. In particular, being understanding and having support was a great force in helping older adults to move through hardship toward positivity. Other findings showed that family connections were a pivotal factor in helping Taiwanese older adults with disabilities to adapt to the life in LTCF. From this study, we found that admission to LTCF had negative impacts on both body and mind for older adults with disabilities, and that individuals could pass through hardship with the compassionate help of others who met their physical and psycho-social needs, showed understanding, paid attention, expressed sympathy, and found ways to get fun and value in life. In taking care of Taiwanese older adults with disabilities newly admitted to LTCF, it is necessary to understand their unwillingness and helplessness to stay there. Needs cannot be understood is an important barrier for older adults with disabilities to adapt life in LTCF. Encouraging family members to visit and provide care that meets the needs as soon as possible and showing sympathy for the older adults with disabilities will help them have better adaption to LTCF life. This study builds on the evidence on the lived experiences of older adults with disabilities newly admitted to LTCFs in Taiwan. As qualitative studies in Taiwan are relatively limited on the topic, we used existing studies from the country as well as the international literature to compare the findings as discussed in the following sections.

For older adults who are disabled and require care, LTCFs are becoming an alternative to the family care model [3,4,5]. New admission to LTCFs is an overwhelming life change for older adults with disabilities [6], having a significant impact on their health and quality of life. In this study, the older adults with disabilities chose to be admitted into the LTCF because they lost the ability to take care of themselves and could not be taken care of at home. It was their common state of mind that living here was the last resort. New admissions to LTCFs tend to have a period of confusion and adaptation, and while learning the standard life processes of the LTCF, they often feel a sense of a transformation to homelessness and may even collapse. However, with the passage of time, the older adults may be able to consider the LTCF as their own place, meaning it feels like home [25].

Older adults are often reluctant to be admitted to LTCFs; however, some older adults will choose to move into LTCFs after becoming disabled. Tsai et al. found that most older adults think that moving to an LTCF is a way to reduce the burden of family care, and family members can continue their commitments without worrying about taking care of them daily [10]. Regardless of whether older adults with disabilities move in LTCFs voluntarily or involuntarily, what is deeply imprinted in their hearts is that living there is a last resort; thus, the way in which to empathize with them, lighten their mood, and help them create their own place in the LTCF as quickly as possible is a problem that professionals should focus on.

In Taiwan’s traditional concept, home is the most anticipated residence for older adults. In this study, the participants described that although their body is in the LTCF, their heart is at home. After older adults with disabilities were admitted into the LTCF, they counted the days every day and looked forward to family visits; for example, some participants described that they like their children bring their favorite food when they come to visit. In Chinese culture, filial piety is a basic virtue and a traditional cultural value. The Confucian creed of filial piety normalizes how children delight and care for their parents. The duties of filial piety include support of, memorializing, compliance with, respect for, love for, and attending to their parents [26]. Confucianism views a person as a part of a family with inter-dependent responsibilities and expectations, and family members are expected to be involved with each other’s lives. The respect for parents has supreme value in the Confucian tradition; if the child is filial, the performance of the parents will be considered good, and parents’ self-esteem is arbitrated by how well they perform as parents [27], which is why the participants expressed the view that they were very happy to be visited and cared by their children.

Life in a LTCF is subject to many restrictions and no autonomy, which can be a problem for the older adults. They described LTCF life as prison-like treatment, where they are deprived of privacy, their own work and rest times are forbidden, they have no freedom to enter and exit the LTCF, and have no control over their lifestyle. These phenomena echoe the common experience and feeling of the life of new residents in the LTCF, meaning a lack of autonomy and personal flexibility [11,13,14]. Although the older adults faced many problems after being admitted to the LTCF, they did understand that, although they did not like it, they still had to live there due to the absence of a better solution, as they were not able to take care of themselves. As Taiwanese older adults grew up in a traditional cultural background, it may be a long struggle for them to treat the LTCF as their home or a place to live for the long term. If older adults can treat the LTCF as a home, they will more easily participate in social activities and integrate into the social environment of the LTCF, which is very beneficial to adjusting to life in the LTCF. However, even if they receive good care, most of older adults do not consider the LTCF as “home”; instead, they will actively change their attitude towards the LTCF, in order that they can adapt better [14]. Staff should deeply consider the impact of cultural values and beliefs on the older adults in Taiwan, pondering over what kind of care measures are more suitable for them in their new stage of living.

Older adults with disabilities that mean are unable to take care of their daily lives will often be admitted to LTCFs. Within their first year in a new LTCF, the psychological pressure has the greatest negative impact, and there is often a decline in their physical functions [3]; thus, new older adult residents living in LTCFs must face many difficulties and challenges. Many older adults mentioned that their needs were not understood in the LTCF and hoped that the staff can take care of them with sympathy, understanding that their limited physical functions must be taken care of for them to live a dignified life. This experience is similar to “retaining the meaning of being alive”, as found in the study of Vaismoradi et al. [28], which expressed the mood of the older adults being cared for in a LTCF. It is hoped that staff can consider individualized human factors in the process of caring for older adults. Moreover, administrative factors affect the way that staff provide individualized care, which often leads to tensions between the older adults and the LTCF. If staff understand the personal lifestyle and cultural needs of the older adults, it will help the new older adult residents in LTCFs to adapt better [4,13]. However, what is more worrying is the influence of traditional Chinese values on the behavior of the older adults, meaning the older adults tend to not fight for their rights, accept what happens as natural, and do not insist on their requirements being met. When faced with something unsatisfactory, they try to explain it as destiny, and are very conservative in the expression of their own needs [29]. It is very difficult for older adults to obtain emotional support in a LTCF, which creates greater obstacles in their adaption to life in LTCF.

Looking forward to receiving emotional support, especially the emotional attachment of family members, is the spiritual chicken soup for older adults beginning to live in LTCFs. Tsai and Tsai found that providing more channels and opportunities for older adults to communicate with family members can increase the richness of their life in the LTCF, as well as their ability to adapt to life there [30]. Having someone in the LTCF to talk with, increasing their social life interactions, and participating in activities are very important parts of emotional support [31], as such things help the older adults to maintain meaningful social relationships in the LTCF. Encouraging older adults to establish new relationships with other residents or staffs in the LTCF, providing them with opportunities to talk about their feeling and experiences, and encouraging their participation in the decision to relocate to the LTCF are helpful for them to adapt to their new life in the LTCF [13]. An important key for the older adults in an LTCF is to enjoy life by maintaining good interpersonal interactions with relatives and friends. Emphasizing the role and autonomy of older adults is important for their happiness and dignity [32]. The older adults and their families agree that visiting is a duty of filial piety, as visits maintain the family relationship with the older adults, ensure the quality of care provided by the LTCF, and allow family members to express their emotional support for the older adults [33]. As LTCFs pay more attention to physical care due to limited capacity, they often ignore the psychological needs of the older adults; thus, it is indeed relatively difficult to achieve family emotional support in an LTCF. The spiritual satisfaction of the older adults after admission to an LTCF is a key factor in life adjustment [34]. It is worth considering how older adults can be helped in order to achieve continuous and uninterrupted emotional communication or contact between family members.

Lee [35] conducted a study in South Korea, finding that new older adult residents can get high emotional and social support from staff in LTCFs. However, this study found that the older individuals were more cautious in getting along with staff in LTCFs. The different results might have been due to the differences in the characteristics of participants between studies. The length of staying in a LTCF was not set as the selection criteria in Lee’s study, while this study interviewed the older adults admitted to the LTCF within 6–9 months. It can be found that new older adult residents require an observation period for them to have an emotional connection with the nursing staff of the LTCF.

While new older adult residents in LTCFs wanted to adapt to the environment, they had not fully understood the people and affairs of the LTCF; thus, they kept silent, sought for good fortune and avoided evil, and reminded themselves to be vigilant in order to deal with life experiences different from the past. They paid attention to their interactions with care personnel, reduced troubles to others, strived to become good residents, and practiced the way of survival. This echoes the findings of Chen and Shyu [27] in that the older adults were used to taking contentment and fate as their main code of conduct, calmly accepting their plight in the new LTCF. Under the influence of such a culture, the older adults described that in order to behave in life in a LTCF, they should always be modest and aware of themselves in order to avoid bad things happening to them, as well as training themselves to become good residents. However, this makes people worry that when new older adult residents in LTCFs encounter problems and fail to take the initiative to raise or report them, it may delay the time to deal with their problems, which can lead to more negative problems or difficulties.

This study found that after the older adults had experienced struggles of staying in the LTCF, they also tried to make themselves better, and they even mentioned that they needed to take care of their health by doing rehabilitation training, as they hoped to return to their homes. This mentality echoes the views of older adults in Taiwan who believe that the LTCF is “a temporary home to nurture health” [36]. Several older adults demonstrated a positive outlook about their life experience of being new residents and appeared to simultaneously regard the LTCF as their own home, but not “home”. They had psychological ambiguities and formed a contradictory relationship in adaptation, which is similar to the mentality of the participants in the study of Nakrem et al. [37]. However, the older adults actively changed their attitude towards the LTCF in order that they could adapt better [14] and worked hard to rehabilitate and improve their body in case they were able to go home one day. This is one of the adaptation methods they practiced in order to not become a burden for caregivers. This confirms the findings of Pardo et al., finding that older adults often struggle with the decline of their physical functions, worry that their decline will restrict their activities in daily living and their independence, fear that they would become completely dependent, and worry about being a burden to others. What they mostly hope for is to be healthy, and some even said they would rather die than rely entirely on others [38]. South Korea also has a strong family-oriented cultural identity, and South Korean older adults also hope not to be a burden to their children [39]. How the older adults identify with their disability is regarded as a unique phenomenon, shaping how they perceive their disabled body and how they interact with the world [40].

In the process of adapting, the older adults evaluated positive and negative situations according to their own life experiences and developed corresponding skills to make themselves better and continue to support themselves to move forward [16]. Similar to the results of Koppitz et al. [3], this study found that the biggest concern of new LTCF residents was to receive care, as receiving care of is one of the modes of adaptation. The older adults felt that the care provided by the LTCF helped them in substance; they identified the advantages of life in an LTCF; and rediscovered the fun and abilities of their original life, which they used to help settle down and develop the kinetic energy required to restart their life. This process is similar to the findings of Heliker and Scholler-Jaquish [25], meaning the older adults were no longer conscious of becoming newly homeless as they learned the ropes and settled in. When the older adults can quickly adapt to a new environment, it will be beneficial to their life in a LTCF. When the older adults in an LTCF can perceive the power of sustenance and support, even if with disabilities and when sick, they can participate in the social affairs of the LTCF, which can help them achieve the goal of active aging [41].

### 4.1. Limitations and Suggestions

In this study, the researchers did their best to consider the age and gender distribution and the time stay of the older adults with disabilities newly admitted to the LTCF. Although the results of the study seemed saturated, they were confined to the participants in two LTCFs in northern Taiwan. We recommend that researchers conduct interviews in other locations, such as southern or eastern Taiwan to ascertain whether geographical factors influence the experiences of newly admitting to LTCFs for older adults with disabilities. In addition, the relationship between older adults, family members, and the staff of LTCFs also affects the experiences of new residents; thus, in future, interviews with family members and staff in LTCFs can be included to increase the richness and breadth of the data.

### 4.2. Implications for Practice

Understanding the perspectives of older adults with disabilities newly admitted to LTCFs is an important step to provide appropriate care for this particular group. The findings of this study have both theoretical and practical value for a fuller understanding of older adults with disabilities newly admitted to LTCFs and can guide staff to provide appropriate interventions to improve quality of care. In practice, staff are able to assist older adults with disabilities adapt life in LTCF by listening their voices to understanding their hardship and care needs, helping them build good relationships and connections with family members, LTCF residents, and others, as well as guiding them to practice the ways to better adapting in LTCF. Staff should gain a deep understanding of older adults’ physical, psychological, and culturally specific care needs by guiding them in sharing their experiences and feelings. Moreover, the findings showed that older adults felt unwillingness and helplessness when newly admitted to LTCF due to the disabilities. It is important for staff to actively care about the mood and feelings and provide appropriate strategies to older adults with disabilities to counteract their hardship and satisfy their current needs. Other findings show that family connections were a pivotal factor, and it is important to involve family families in the care of Taiwanese older adults with disabilities. Staff should encourage family members to visit often and show their care and support to the older adults. Staff also should have highly sensitive observation skills, evaluate the older adults’ emotional state, encourage them to express their feelings, lead them to express negative sentiments, and provide them with positive feedback. Finally, staff can help older adults develop strategies to survive and adapt better in LTCFs, taking into account individual care needs established on the basis of discussions with the older adults themselves.

## 5. Conclusions

As older adults with disabilities were admitted to the LTCF due to insufficient self-care ability and family member’s difficulty in taking care of them, they think that living in a LTCF is a last resort. The older adults with disabilities expected being taken care of in the LTCF attentively and their needs being understood, and they looked forward to receiving emotional support. As time went by, they realized that although they do not like it, they will continue to live in the LTCF; thus, they used self-adjustment and established friendly relationships with the staff of the LTCF and assumed a silent and vigilant attitude to live safe and sound in the LTCF. In order to find their focus and value of life and restart their living activities, the older adults took good care of their bodies through rehabilitation. This study focused on traditional Chinese culture in the older adults, as older adults tend to emphasize home and worry about the burden on their shoulders and their responsibility to their families; however, such characteristics also make it difficult for older adults to adapt to their new life in a LTCF. When the older adults with disabilities are newly admitted to a LTCF, we must pay attention to their care needs and consider the life adaptation problems accompanying their new life in the LTCF in order to assist them to open up to new life and enrich their daily lives while living in a LTCF.

## Figures and Tables

**Table 1:** Interview guidelines.

Number	Guidance Questions
1	Under what circumstances did you come to live here?
2	Could you please talk about your life in the long-term care facility? (1) How do you arrange your daily life here? (2) What activities have you participated in? (3) How are you getting along with other residents?
3	What do you think the long-term care facility makes you most like and least like? (What do you think about your most favorited and least favorited thing here? Please describe.)
4	How does it feel to live here?
5	What problems or difficulties did you encounter after moving into the long-term care facility? (1) How did you feel? (2) How did you deal with it?
6	Apart from what you just expressed, is there anything else that you want to share with me?

**Table 2:** Basic information of the participants.

Demographics	N	%
Age		
65–70	4	26.7
71–80	9	60.0
81–90	2	13.3
Gender		
Male	8	53.3
Female	7	46.7
ADL score		
35–50	7	46.7
55–50	8	53.3
Months of stay in LTCFs		
3–4	3	20.0
5–6	8	53.3
7–9	4	26.7
Reason for admission: disability due to		
Stroke	4	26.7
Falls	7	46.7
Dialysis + foot injury	2	13.3
Work injury	2	13.3

**Table 3:** Themes and sub-themes of the lived experiences of newly admitted to LTCFs among older adults with disabilities (*n* = 15).

Theme	Sub-Theme	Participants with Experience		
		*n*	%	Name Code
Living here is a last resort	(1) Difficulty being cared for at home	15	100.0	A, B, C, D, E, F, G, H, I, J, K, L, M, N, O
	(2) Hard to get home	9	60.0	A, B, D, G, H, I, L, M, N
2. Don’t like it here but have to stay	(1) Life here is like in a prison	7	46.7	A, B, D, F, H, M, O
	(2) The body here but the heart is not	8	53.3	A, B, D, F, H, L, M, O
	(3) Living here helplessly	8	53.3	A, B, D, F, H, L, M, O
3. Needs not understood	(1) Demand cannot be met immediately	8	53.3	C, D, E, F, G, H, M, N
	(2) Hope to be cared for with sympathy	11	73.3	B, D, E, F, G, H, J, L, M, N, O
4. Hope for emotional support	(1) Love family to visit	11	73.3	A, C, D, E, G, H, I, J, L, M, N
	(2) Like to be accompanied	9	60.0	C, D, E, G, I, J, L, M, N
	(3) Looking forward to building relationships with residents	12	80.0	A, B, C, D, F, G, H, J, K, L, M, N
5. Practicing the way of survival	(1) Staying alert and remaining silent	7	46.7	C, D, G, J, K, N, O
	(2) Trying to have fun in life	7	46.7	A, C, E, H, J, K, O
	(3) Being a good resident	10	66.7	C, D, E, G, H, J, K, L, N, O
6. Striving to be better	(1) Finding a way to keep the health from going bad	8	53.3	B, E, G, H, I, J, K, L
	(2) Participating in active rehabilitation for progress	9	60.0	B, F, G, H, I, J, L, M, O
	(3) Trying to find the focus and value of life	8	53.3	A, C, E, G, K, L, M, O,

## Data Availability

The data presented in this study are available on request from the corresponding author. The data are not publicly available due to privacy.
